# Detection of telomerase activity in peritoneal lavage fluid from patients with gastric cancer using immunomagnetic beads

**DOI:** 10.1054/bjoc.2000.1408

**Published:** 2000-10

**Authors:** N Mori, M Oka, S Hazama, N Iizuka, K Yamamoto, S Yoshino, A Tangoku, T Noma, K Hirose

**Affiliations:** Departments of Surgery II, Biochemistry II, Yamaguchi University School of Medicine, 1–1–1 Minamikogushi, Ube, Yamaguchi, 755–8505; Biomedical Research Institute, Kureha Chemical Industry Co Ltd, 3–26–, 2Hyakunin-cho, Tokyo, Shinjuku-ku, 169, Japan

**Keywords:** gastric cancer, peritoneal lavage, telomeric repeat amplification protocol assay, immunomagnetic beads

## Abstract

Cytologic examination of peritoneal lavage fluid is a useful predictor of peritoneal recurrence in gastric cancer. However, this technique is not overly sensitive and requires special abilities in the cytologist. In this study, telomerase activity was used to detect free cancer cells in peritoneal lavage fluid from patients with gastric cancer. In the first part, 12 lavage-fluid samples obtained from 12 patients with gastric cancer were analysed using the conventional telomeric repeat amplification protocol (TRAP) assay. Three of five patients with early gastric cancer had positive telomerase activity. These false-positive results may have been due to lymphocyte contamination. Furthermore, polymerase chain reaction inhibitors were also detected in the lavage-fluid samples. Therefore, we developed a novel method for elimination of haematopoietic cell and Taq polymerase inhibitors to increase the accuracy of the TRAP assay using immunomagnetic beads, which bind to most normal and neoplastic human epithelial cells. Telomerase activity was found in 10 of 20 (50%) lavage-fluid samples from patients with serosal or subserosal invasion. Cytologic examination was positive in nine of 20 (45%) samples. Both the telomerase activity and cytology were negative in all 14 patients without serosal or subserosal invasion. These results suggest that the TRAP assay combined with immunomagnetic beads might be useful for detection of free cancer cells in the peritoneal space in gastric cancer without the aid of an experienced cytologist. © 2000 Cancer Research Campaign


*British Journal of Cancer* (2000) **83**(8), 1026–1032
© 2000 Cancer Research Campaign
doi: 10.1054/ bjoc.2000.1408, available online at http://www.idealibrary.com on IDEAL®

### Detection of telomerase activity in peritoneal lavage fluid from patients with gastric cancer using immunomagnetic beads

**N Mori[1], M Oka[1], S Hazama[1], N Iizuka[1], K Yamamoto[1], S Yoshino[1], A Tangoku[1], T Noma[2] and K Hirose[3]**

[1]Departments of [1]Surgery II, and [2]Biochemistry II, Yamaguchi University School of Medicine, 1–1–1 Minamikogushi, Ube, Yamaguchi 755–8505; [3]Biomedical Research Institute, Kureha Chemical Industry Co Ltd, 3–26–2, Hyakunin-cho, Shinjuku-ku, Tokyo 169, Japan

**Summary** Cytologic examination of peritoneal lavage fluid is a useful predictor of peritoneal recurrence in gastric cancer. However, this technique is not overly sensitive and requires special abilities in the cytologist. In this study, telomerase activity was used to detect free cancer cells in peritoneal lavage fluid from patients with gastric cancer. In the first part, 12 lavage-fluid samples obtained from 12 patients with gastric cancer were analysed using the conventional telomeric repeat amplification protocol (TRAP) assay. Three of five patients with early gastric cancer had positive telomerase activity. These false-positive results may have been due to lymphocyte contamination. Furthermore, polymerase chain reaction inhibitors were also detected in the lavage-fluid samples. Therefore, we developed a novel method for elimination of haematopoietic cell and Taq polymerase inhibitors to increase the accuracy of the TRAP assay using immunomagnetic beads, which bind to most normal and neoplastic human epithelial cells. Telomerase activity was found in 10 of 20 (50%) lavage-fluid samples from patients with serosal or subserosal invasion. Cytologic examination was positive in nine of 20 (45%) samples. Both the telomerase activity and cytology were negative in all 14 patients without serosal or subserosal invasion. These results suggest that the TRAP assay combined with immunomagnetic beads might be useful for detection of free cancer cells in the peritoneal space in gastric cancer without the aid of an experienced cytologist. © 2000 Cancer Research Campaign

**Keywords**: gastric cancer; peritoneal lavage; telomeric repeat amplification protocol assay; immunomagnetic beads

Gastric cancer is a common malignancy in humans (Dupont et al, 1978; Koga et al, 1984; Kitamura et al, 1995). Although significant progress has been made in the early detection and surgical treatment of gastric cancer, tumours invading the gastric serosa still carry a poor prognosis, with a 5-year survival of 20–40% (Kodama et al, 1981; Bizer, 1983; Baba et al, 1989). The most common mode of postoperative recurrence is peritoneal dissemination, which may occur by release of free cancer cells into the peritoneal space (Iitsuka et al, 1979; Nakanishi et al, 1997). It has been reported that patients with free cancer cells in the peritoneal cavity at the time of surgery have a considerably worse prognosis than those who do not (Koga et al, 1984; Bonenkamp et al, 1996; Schott et al, 1998). The detection of free cancer cells is therefore considered as an important predictor of peritoneal recurrence in gastric cancer.

Cytologic examination of lavage fluid is the conventional method to detect free cancer cells in the peritoneal space. A close association has been demonstrated between a positive result and low survival rates (Koga et al, 1984; Bonenkamp et al, 1996; Schott et al, 1998). However, the sensitivity of cytology is relatively low. Abe et al (1995) have reported patients with negative cytology who subsequently developed peritoneal dissemination. Therefore, it was necessary to develop a more sensitive method to detect free cancer cells in the peritoneal space.

*Received 28 July 1999*
*Revised 31 May 2000*
*Accepted 27 June 2000*

*Correspondence to:* M Oka

Telomerase is the ribonucleoprotein that synthesizes the strand of telomeric DNA (Kim et al, 1994; Rhyu, 1995). In all normal somatic cells, the chromosomes lose about 50–200 nucleotides of telomeric sequence per cell division because of the inability of DNA polymerase to replicate the end of linear DNA (Watson, 1972). After a certain number of cell divisions, the cells eventually stop dividing and reach the senescence stage. This phenomenon is thought to be the mitotic clock by which cells count their own divisions and recognize their life-span (Harley, 1991). In contrast to normal somatic cells, immortal cells preserve their telomere length in spite of indefinite division, by the action of the enzyme telomerase. Therefore, the expression of telomerase is believed to play an important role in immortalization and carcinogenesis.

Recently, an improved method to measure telomerase activity (telomeric repeat amplification protocol (TRAP) assay) was developed (Kim et al, 1994). High telomerase activity has been reported in a variety of cancer cells (Kim et al, 1994; Hiyama et al, 1995*a*; 1997; Langford et al, 1995; Tahara et al, 1995*a*; Saji et al, 1997). Telomerase activity has been detected in 85–89% of gastric cancers (Hiyama et al, 1995*b*; Tahara et al, 1995*b*; Ahn et al, 1997). It is absent in most adult somatic cells. Thus, telomerase activity is considered a useful diagnostic marker for malignant tumours. On the other hand, telomerase activity has been detected in haematopoietic cells such as lymphocytes (Hiyama et al, 1995*c*; Counter et al, 1995; Bodnar et al, 1996). The high sensitivity of this assay could result in false-positives, reducing the specificity of this test (Yashima et al, 1997). This may be particularly important in the detection of a small number of cancer cells.

Furthermore, it has also been reported that tissue or cell extracts containing Taq polymerase inhibitors could interfere with the

TRAP assay (Wright et al, 1995), resulting in false-negative results. Exclusion of contamination by haematopoietic cells and Taq polymerase inhibitors is therefore required to accurately evaluate telomerase activity.

In the present study, we developed a novel method for the elimination of haematopoietic cells and Taq polymerase inhibitors to increase the accuracy of the TRAP assay, using immunomagnetic beads (IMB). Telomerase activity was measured in peritoneal lavage fluid from patients with gastric cancer using this modified technique.

### MATERIALS AND METHODS

### Patients

We studied 46 patients with gastric cancer admitted to our department between 1996 and 1998 (Table 1). All 46 had a primary adenocarcinoma of the stomach, and no synchronous or metachronous carcinomas. Prior to surgery, 44 patients received no anticancer therapy. The other two patients received 5-fluorouracil and cisplatin. The 32 men and 14 women had ages ranging from 32–84 years (mean 61.7 years). A conventional assay of telomerase activity was performed in 12 of the 46 patients. The modified assay with immunomagnetic beads was performed in 34 patients. The operative and microscopic findings were described according to the criteria of the International Union Against Cancer (UICC) TNM Classification (Hermanek et al, 1987). Microscopic tumour depth was described as mucosa (m), submucosa (sm), muscularis propria (mp), subserosa (ss) and serosa (s). Informed consent was obtained from all patients. This study was approved by the Institutional Human Investigation Committee of Yamaguchi University School of Medicine.

### Peritoneal lavage fluid

After laparotomy, 100 ml of saline solution was poured into the upper abdominal cavity, which contained the primary lesion. It was collected in a heparinized syringe. Half of each lavage-fluid sample was examined by cytology. Cytology samples were stained by Papanicolaou and Giemsa. The remaining fluid was centrifuged at 250 ***g*** for 10 min. The pelleted samples were washed twice with calcium-free, magnesium-free, phosphate-buffered saline (PBS(–)). The pellets were then stored in cell freezing preservation liquid (Cell Banker®; Nippon-zenyakukougyo Co Ltd, Fukushima, Japan) at –80°C until assay.

### Peripheral blood mononuclear cell preparation

Peripheral blood samples were collected aseptically from two healthy volunteers using heparin anticoagulant. Peripheral blood mononuclear cells (PBMCs) were obtained as described previously (Oka et al, 1996) and stored as described above until assay.

### Cell line

Human gastric signet-ring cell carcinoma cell line KATO-III was obtained from the Japanese Cancer Research Resources Bank (Tokyo). This cell line was maintained in Rosewall Park Memorial Institute (RPMI)-1640 medium (GIBCO, New York, USA) supplemented with L-glutamine and 10% heat-inactivated fetal bovine serum in a 5% $CO_2$ atmosphere at 37°C.

### Telomeric repeat amplification protocol (TRAP) assay

Telomerase activity in the cell lysates was measured according to the TRAP method described by Kim et al (1994). Briefly, samples and cultured cells were pelleted (250 ***g*** for 10 min) in PBS (–), and the supernatant was removed. Then, the pellets were suspended using micropipetter in 100–200 µl of ice-cold lysis buffer (10 mM Tris-HCl, pH 7.5, 1 mM $MgCl_2$, 1 mM ethylenebis (oxyethylene-nitrilo)-tetraacetic acid (EGTA), 0.1 mM phenylmethylsulfonyl fluoride (PMSF), 5 mM 2-mercaptoethanol, 0.5% 3-((3 cho-amidopropyl)dimethylammonio)-1-propanesulphate (CHAPS) and 10% glycerol), and incubated on ice for 30 min. Extracts were centrifuged at 12 000 ***g*** for 20 min at 4°C. An 80–160 µl aliquot of the supernatant was collected, flash-frozen in liquid nitrogen, and stored at –80°C. The protein concentration was adjusted after measurement by the Bladford method (Protein Assay Kit; Bio-Rad Laboratories, Richmond, CA, USA). Before the polymerase chain reaction (PCR), 0.1 µg of CX primer (5′-CCCTTACCCTTACC-CTTACCCTAA-3′) was lyophilized in a 0.5-ml tube (Sci-media Ltd, Tokyo, Japan). After sealing the CX primer by heating at 90°C for 30 s, 50 µl of TRAP reaction buffer containing 50 mM Tris-HCl (pH 8.9), 1.5 mM $MgCl_2$, 50 mM potassium chloride (KCl), 0.05% Tween 20 (Sigma Chemical Co, Tokyo, Japan), 1 mM EGTA, 0.1 µg of TS oligonucleotide (5′-AATCCGTC-GAGCAGAGTT-3′), 50 µM of each deoxynucleotide triphosphate, 1 µg of T4-gene 32 protein (Boehringer Mannheim Corp, Tokyo, Japan), 2 U of Taq DNA polymerase (Ampli-Taq; Perkin Elmer-Cetus, Norwalk, CT, USA), 3 µl of appropriately diluted CHAPS cell extract, and 2 µCi of [$\alpha$-$^{32}$P] deoxycytidine triphosphate (Amersham, Tokyo, Japan) was added onto a solidified wax (Ampli-Wax; Perkin Elmer-Cetus). Then, tubes were set in a thermal cycler (model PC-700; ASTEC, Fukuoka, Japan), and telomere elongation was conducted for 30 min at 22°C. PCR amplification was achieved with 33 cycles of incubation at 94°C for 30 s, 50°C for 30 s, and 72°C for 45 s. Aliquots (20 µl) of radiolabelled PCR products were separated using 10% polyacrylamide gel electrophoresis. The gels were dried and exposed to a Phosphor Imaging Plate (Fuji Co Ltd, Tokyo, Japan), and visualized with the use of a BAS 2000 image analyser (Fuji Co). For positive and negative controls, 0.3 µg of extract of KATO-III, and 0.3 µl of lysis buffer were used, respectively, on every assay. Detectable telomerase activity was defined as a hexanucleotide ladder of three or more bands not present in the matched negative controls (Kim et al, 1994; Saji et al, 1997).

### TRAP assay in combination with immunomagnetic beads

In order to isolate the cancer cells and remove the inhibitors in lavage fluid, immunomagnetic beads (IMB; Dynabeads® anti-Epithelial Cell; DYNAL Inc, Lake Success, NY, USA) were used. The beads are magnetizable polystyrene coated with a mouse IgG-1 monoclonal antibody (mAb Ber-EP4) specific for two (34 and 39 kDa) glycopolypeptide membrane antigens expressed on most normal and neoplastic human epithelial tissues. Samples were resuspended in 5 ml of PBS (–), $5 \times 10^7$ IMB was added, and the mixture was incubated for 60 min at 4°C. Then, the tubes were

© 2000 Cancer Research Campaign 

**Table 1** Clinicopathologic features of patients with gastric cancer, and telomerase activity in lavage-fluid samples

| Patient no. | Age/Sex | Histology[a] | Depth of Invasion[b] | pN[a] | pM[a] | stage[a] | Cytology | Telomerase activity without IMB[a] | Telomerase activity with IMB[a] |
|---|---|---|---|---|---|---|---|---|---|
| 1 | 71/M | G1 | sm | 0 | 0 | Ia | I | Negative | |
| 2 | 56/M | G1 | sm | 0 | 0 | Ia | I | Negative | |
| 3 | 32/F | G3 | sm | 0 | 0 | Ia | I | Positive | |
| 4 | 54/M | G1 | sm | 0 | 0 | Ia | I | Positive | |
| 5 | 51/M | G1 | sm | 0 | 0 | Ia | I | Positive | |
| 6 | 39/M | G3 | mp | 1 | 0 | II | I | Negative | |
| 7 | 81/M | G3 | ss | 2 | 0 | III | II | Positive | |
| 8 | 80/M | G2 | ss | 1 | 0 | II | II | Positive | |
| 9 | 47/F | G1 | s | 0 | 0 | II | I | Positive | |
| 10 | 71/M | G3 | s | 2 | 1 PER | IV | I | Positive | |
| 11 | 77/F | GX | s | N2 | 1 PER | IV | I | Positive | |
| 12 | 53/F | G3 | s | 2 | 1 PER | IV | V | Positive | |
| 13 | 48/M | G2 | m | 0 | 0 | Ia | I | | Negative |
| 14 | 79/M | G3 | m | 0 | 0 | Ia | I | | Negative |
| 15 | 48/M | G1 | m | 0 | 0 | Ia | I | | Negative |
| 16 | 80/M | G1 | sm | 1 | 0 | Ib | I | | Negative |
| 17 | 37/M | G2 | sm | 0 | 0 | Ia | I | | Negative |
| 18 | 72/F | G2 | sm | 1 | 0 | Ib | I | | Negative |
| 19 | 61/F | G2 | sm | 1 | 0 | Ib | I | | Negative |
| 20 | 74/F | G3 | sm | 0 | 0 | Ia | I | | Negative |
| 21 | 58/M | G3 | sm | 0 | 0 | Ia | I | | Negative |
| 22 | 57/M | G2 | mp | 0 | 0 | Ib | I | | Negative |
| 23 | 69/M | G2 | mp | 1 | 0 | II | I | | Negative |
| 24 | 50/M | G3 | mp | 2 | 0 | IIIa | I | | Negative |
| 25 | 78/M | G3 | mp | 0 | 0 | Ib | I | | Negative |
| 26 | 72/M | G3 | mp | 1 | 0 | II | I | | Negative |
| 27 | 69/M | G2 | ss | 1 | 0 | II | I | | Negative |
| 28 | 70/M | G3 | ss | 2 | 0 | IIIa | I | | Negative |
| 29 | 75/F | G2 | ss | 2 | 0 | IIIa | II | | Negative |
| 30 | 71/M | G2 | ss | 1 | 0 | II | II | | Negative |
| 31 | 68/M | G2 | ss | 0 | 0 | Ib | II | | Negative |
| 32 | 52/F | G3 | ss | 2 | 0 | IIIa | II | | Negative |
| 33 | 62/M | G2 | ss | 1 | 0 | II | II | | Negative |
| 34 | 55/M | G3 | ss | 1 | 0 | II | II | | Positive |
| 35 | 49/M | G3 | ss | 2 | 0 | IIIa | V | | Positive |
| 36 | 58/M | G2 | s | 0 | 0 | II | II | | Negative |
| 37 | 62/M | G3 | s | 2 | 0 | IIIb | II | | Negative |
| 38 | 68/F | G2 | s | 0 | 0 | II | II | | Positive |
| 39 | 82/M | G3 | s | 1 | 0 | IIIa | V | | Negative |
| 40 | 84/M | G2 | s | 1 | 0 | IIIa | V | | Positive |
| 42 | 77/M | G1 | s | 1 | 0 | IIIa | V | | Positive |
| 41 | 69/F | G3 | s | 2 | 0 | IIIb | V | | Positive |
| 43 | 62/F | G2 | s | 2 | 0 | IIIb | V | | Positive |
| 44 | 53/F | G3 | s | 2 | 1 PER | IV | V | | Positive |
| 45 | 71/M | G3 | s | 1 | 1 PER | IV | V | | Positive |
| 46 | 70/F | G3 | s | 2 | 1 PER | IV | V | | Positive |

[a] According to the criteria of the International Union Against Cancer TNM Classification; [b] m = mucoa; sm = submucosa; mp = muscularis propria; ss = subserosa; s = serosa

© 2000 Cancer Research Campaign placed on a magnetic device (Dynal MPC®; DYNAL Inc) for 2 min, and the fluid was pipetted off. After the tubes were removed from the magnetic device, the material with the beads were resuspended in PBS(–) and centrifuged at 250 ***g*** for 10 min, and the supernatant was removed. The pellets were resuspended in 50 µl of ice-cold lysis buffer and centrifuged at 12 000 ***g*** for 20 min at 4°C. Then, 30 µl of the supernatant was collected. The TRAP assay was performed.

### RESULTS

### Conventional TRAP assay

In the first study, 12 lavage-fluid samples were examined using the conventional TRAP assay. Telomerase activity was detected in nine of 12 (75%) samples. Of these, three were from patients with tumour invasion to the submucosa (Figure 1). These fluid samples, which were cytologically negative, contained a number of lymphocytes, erythrocytes, granulocytes, and mesothelial cells. Telomerase activity was also detected in the ascites obtained from a patient with liver cirrhosis, which contained a number of PBMCs but not cancer cells. In several samples, we counted the number of PBMCs. We founded $1 \times 10^6$–$1 \times 10^7$ of PBMCs in these samples. Therefore, we subsequently evaluated telomerase activity of peripheral blood mononuclear cells (PBMCs).

PBMCs were obtained from healthy volunteers, and telomerase assays were performed on extracts containing $1 \times 10^3$, $3 \times 10^3$, $1 \times 10^4$ and $1 \times 10^5$ cells. Telomerase activity was detected in the $1 \times 10^4$ PBMC samples, which were not stimulated. Thus the false-positive results in the lavage-fluid and ascites samples were thought to be due to contamination by haematopoietic cells such as lymphocytes.

Furthermore, it has been shown that tissue or cell extracts containing Taq polymerase inhibitors can cause false-negative results (Wright et al, 1995). To evaluate this possibility, extracts from 10 lavage-fluid samples were mixed with the same volume of extracts from the KATO-III cells. The mixtures were analysed using the TRAP assay. Four of 10 extracts inhibited the TRAP signals of the cell line extracts. In these four samples, the concentrations of protein ranged from 14.9–20.9 µg $\mu l^{-1}$ (mean 17.98 µg $\mu l^{-1}$). The mean concentration in the other six samples was 4.42 µg $\mu l^{-1}$ (Figure 2). Furthermore, extracts from KATO-III cells mixed with 200 µl of whole blood, which was frequently included in the lavage fluid, were analysed. The ladder was weaker than in the extracts from identical numbers of KATO-III cells (Figure 3). These results strongly suggested that contaminated protein could inhibit the TRAP assay.

### Novel TRAP assay with immunomagnetic beads

To remove the lymphocytes and Taq polymerase inhibitors, we attempted to isolate the tumour cells from the other components in the lavage fluid before protein extraction, using Immunomagnetic beads (IMB). First, telomerase activity was compared using the conventional TRAP assay and the novel TRAP assay with IMB. The conventional assay detected telomerase activity in the extracts from $1 \times 10^4$ PBMCs. The novel TRAP assay with IMB did not detect telomerase activity even in the extracts from $1 \times 10^5$ PBMCs (Figure 3). The TRAP assay with IMB detected telomerase activity in the extracts from 10 KATO-III cells. The signal intensity was identical to that without IMB. The extract from the IMB alone showed no signal (Figure 3). Whole blood cells inhibited the signal intensity of the KATO-III cells. By removing the whole blood cells from the KATO-III cells using IMB, the original ladder was detected (Figure 3).

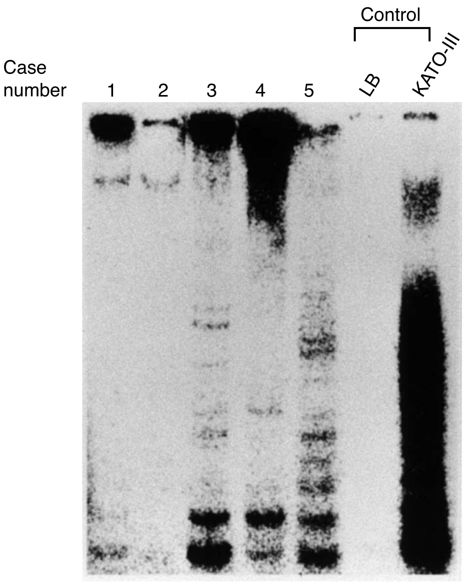

**Figure 1** Telomerase activity in peritoneal lavage-fluid samples in patients with early gastric cancer using the conventional telomeric repeat amplification protocol (TRAP) assay. Numbers above Lanes are case numbers. Lysis buffer (LB) and extracts from the human gastric signet-ring cell carcinoma cell line (KATO-III) were used as controls. Cases 3, 4, and 5 showed 6-base pair ladder signals. In all patients, the depth of invasion was submucosa

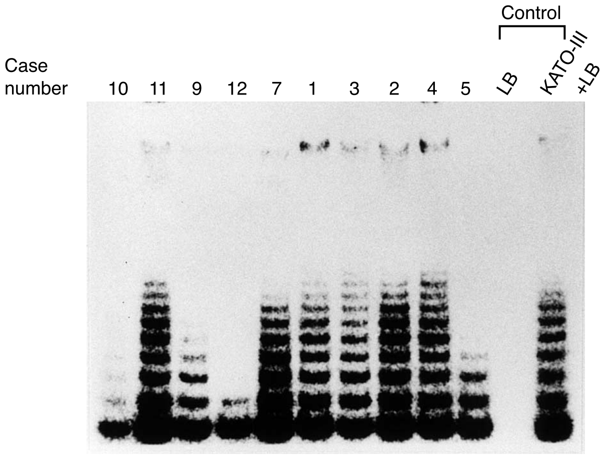

**Figure 2** Evaluation of the existence of Taq polymerase inhibitors in lavage-fluid samples. Extracts from samples were mixed with the same volume of extracts from KATO-III cells (0.44 µg $\mu l^{-1}$). The mixtures were analysed by the TRAP assay. Cases 5, 9, 10, and 12 had inhibitors against the TRAP assay

*© 2000 Cancer Research Campaign*

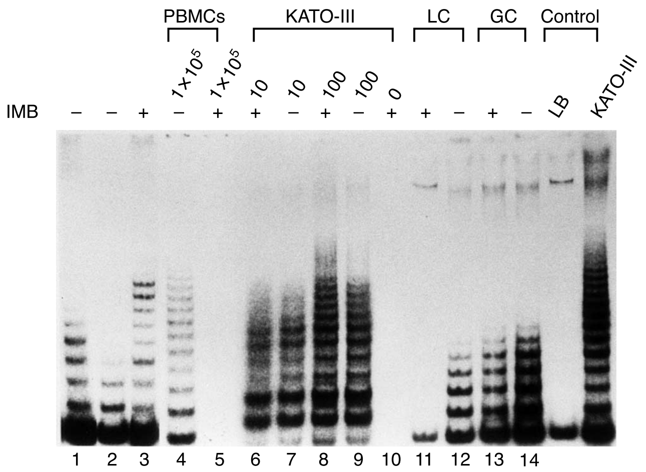

**Figure 3** Telomerase activity with (+) or without (–) immunomagnetic beads (IMB) pretreatment Lane 1, $1 \times 10^4$ KATO-III cells alone; Lane 2, $1 \times 10^4$ KATO-III cells mixed with 200 μl of whole blood without IMB: Lane 3, $1 \times 10^4$ KATO-III cells mixed with 200 μl of whole blood with IMB. The ladder signal in Lane 2 was weak compared to Lane 1. The signal in Lane 3 was almost identical to that in Lane 1. Telomerase activity was detected in the extract from $1 \times 10^5$ PBMCs (Lane 4), but not in the extract from $1 \times 10^5$ PBMCs treated with IMB (Lane 5). There was no difference in ladder intensity of KATO-III treated with or without IMB (Lanes 6–9). The extract from IMB alone showed no signal (Lane 10). LC = ascites from a patient with liver cirrhosis, GC = ascites from a patient with gastric cancer and positive cytology. Conventional TRAP assay (without IMB) showed a clear ladder in both LC and GC samples (Lanes 12 and 14). The TRAP assay with IMB showed a ladder in the GC sample (Lane 13), but not the LC sample (Lane 11)

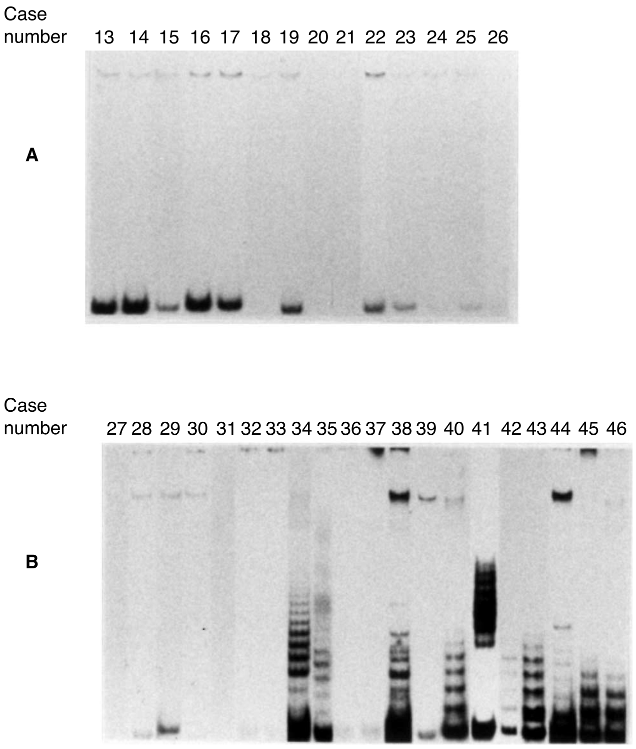

**Figure 4** Telomerase activity in lavage-fluid samples using TRAP assay with IMB. (**A**) None (0%) of the 13 patients without subserosal or serosal invasion demonstrated telomerase activity. (**B**) Nine (60%) of the 15 samples from patients with serosal or subserosal invasion showed a positive ladder. Cytologic examination was positive in seven (46.7%) of the 15 samples

Telomerase activity in the ascites from the patient with liver cirrhosis was negative by TRAP assay with IMB, but positive by TRAP assay without IMB. The same analysis, with and without IMB, was carried out using ascites from a gastric cancer patient with positive cytology. Clear ladders were detected in both assays (Figure 3).

The TRAP assay with IMB revealed 10 positives (29.4%) among 34 lavage fluid samples. The positives were recorded according to depth (see Materials and Methods) of cancer invasion: zero of three (0%) m, zero of six (0%) sm, zero of five (0%) mp, two of nine (22.2%) ss, and eight of 11 (72.7%) s (Figure 4). Telomerase activity in the lavage fluid was detected in none (0%) of the 14 patients without subserosal or serosal invasion. It was found in 10 (50%) of the 20 patients with serosal or subserosal invasion. Cytologic examination revealed nine positives (26.5%) among 34 lavage fluid samples. Telomerase activity was positive in eight of nine patients with positive cytology. Telomerase activity was also detected in two patients with negative cytology (depth ss and s, respectively).

### DISCUSSION

In patients with advanced gastric cancer invading as far as the gastric serosa, the 5-year survival rate has been reported to range from 20–40% (Kodama et al, 1981; Bizer, 1983; Baba et al, 1989; Kodera et al, 1996). In particular, patients with serosal involvement or positive lavage-fluid cytology carry an even worse prognosis (Koga et al, 1984; Bonenkamp et al, 1996; Schott et al, 1998). Peritoneal dissemination is a significant cause of mortality after surgery for gastric cancer. This mode of recurrence may be caused by cancer cells already in place at the time of surgery (Iitsuka et al, 1979; Nakanishi et al, 1997). Therefore, it is important for staging and follow-up to determine whether cancer cells exist in the peritoneal space.

Cytologic examination of lavage fluid is a useful method for detecting cancer cells. However, the sensitivity is relatively low, and the reliability of morphologic diagnosis is limited. For example, it is difficult to distinguish between benign reactive mesothelial cells and well-differentiated carcinoma cells (Schofield et al, 1997). In fact, peritoneal dissemination has been detected in patients with negative cytology (Abe et al, 1995).

Recently, high telomerase activity has been reported in a variety of cancer cells (Kim et al, 1994; Hiyama et al, 1995*a*; 1997; Langford et al, 1995; Tahara et al, 1995*a*; Saji et al, 1997) including gastric cancer (Hiyama et al, 1995*b*; Tahara et al, 1995*b*; Ahn et al, 1997). It is notably absent in most somatic cells. Thus, telomerase activity can be used as a marker for malignant tumours. However, the enzyme is not completely specific for malignancy. For instance, lymphocytes are known as a source of telomerase activity (Counter et al, 1995; Hiyama et al, 1995*c*; Bodnar et al, 1996). The development of the TRAP assay has enabled the detection of even the very weak activity in lymphocytes and small numbers of tumour cells. Counter et al (1995) have reported that, unlike other somatic tissues, peripheral, cord-blood, and bone-marrow leukocytes from normal donors express low levels of

 *© 2000 Cancer Research Campaign*

telomerase activity. Hiyama et al (1995*c*) have reported that 55 (44.4%) of 124 individuals exhibited telomerase activity in samples from $1 \times 10^4$ PBMCs. In our study, the conventional TRAP assay demonstrated telomerase activity in three of five lavage fluid samples obtained from patients with tumour invasion to the submucosa, and one ascites sample obtained from a patient with liver cirrhosis. In these samples, cytologic examination showed a number of lymphocytes, but no tumour cells. The conventional TRAP assay in samples from $1 \times 10^4$ PBMCs also showed a clear ladder. These data demonstrated that contaminated lymphocytes can affect the interpretation of the TRAP assay in a small number of cells.

It has also been reported that inhibitors of Taq polymerase can yield a false-negative result (Wright et al, 1995). The source of Taq polymerase inhibitors is still unclear.

Hiyama et al (1995*b*) have reported that extracts of gastric cancer but normal gastric mucosa contain the inhibitors. Piatyszek et al (1995) have reported that inhibitors can be seen in extracts from normal human tissues such as brain, intestine, kidney, liver, lung, spleen, and adrenal gland. In the present study, we also observed inhibitors of the TRAP assay in extracts obtained from lavage-fluid samples from patients with early gastric cancer, and in extracts of whole blood. Protein extracted from erythrocytes, granulocytes, mesothelial cells, and lymphocytes may inhibit the TRAP assay.

We therefore used IMB to remove both lymphocytes and inhibitors before extraction of protein from lavage-fluid samples. The beads were coated with a mouse IgG-1 monoclonal antibody (Ber-EP4) specific for antigens expressed on most normal and neoplastic human epithelial cells including gastric cancer cells, but not on non-epithelial tissue such as erythrocytes, granulocytes, mesothelial cells, and lymphocytes (Latza et al, 1990). It has been reported that Ber-EP4 reactivities for adenocarcinomas are more than 80% (Ordonez, 1998), and its reactivities for gastric cancers are 100% (Latza et al, 1990; Gaffey et al, 1992).

The efficacy of IMB in the TRAP assay was evaluated using KATO-III cells, PBMCs, ascites, and whole blood. Telomerase activity was detected in the KATO-III cells without loss of activity. This method resolved not only the false-positive results due to the lymphocytes, but also the false-negative results due to the inhibitors. This technique is clearly superior to the conventional TRAP assay.

The TRAP assay with IMB demonstrated telomerase activity in 10 samples. The cytology was positive in nine samples. Telomerase activity was found in eight of nine patients with positive cytology. In addition, two patients with negative cytology had a positive TRAP assay with IMB. Although the depth of tumour invasion in one of these two patients was only to the subserosa, telomerase activity suggested the existence of cancer cells in the abdominal cavity. It was suspected that direct serosal invasion might have been overlooked at pathologic examination, or that cancer cells penetrated the stomach wall.

In two cases with negative cytology and with positive result of TRAP assay one was dead 3 months after surgery with lymphnode recurrence but without clear evidence of peritoneal recurrence, and the other received anticancer therapy after surgery and was alive without recurrence for 2 years. One case with positive cytology, but negative with the TRAP assay, was dead with peritoneal recurrence 69 days after resection.

This study enlightens the study of conventional TRAP assay of cancer cells in the peritoneal space. Although the positive rate of TRAP assay with IMB is not very significantly superior to cytologic examination, this new method is sufficiently sensitive and completely objective. Therefore, this method may play a role in aiding assessment by identifying possible false-negative samples on routine cytological examination. It is considered that the cases with free cancer cells in the peritoneal cavity, if not with peritoneal dissemination, carry a poor prognosis (Bando et al, 1999; Kodera et al, 1999; Suzuki et al, 1999). Therefore, adjuvant therapy should be attempted in such patients in the future. Since the follow-up in this study was short, the significance of positive telomerase activity in terms of prognosis cannot be determined. Long-term observation would be required.

### ACKNOWLEDGEMENTS

This work was supported in part by a Grant-in-Aid from the Ministry of Education, Science and Culture of Japan.

### REFERENCES

Abe S, Yoshimura H, Tabara H, Tachibana M, Monden N, Nakamura T and Nagaoka S (1995) Curative resection of gastric cancer: limitation of peritoneal lavage cytology in predicting the outcome. *J Surg Oncol* **59**: 226–229

Ahn MJ, Noh YH, Lee YS, Lee JH, Chung TJ, Kim IS, Choi IY, Kim SH, Lee JS and Lee KH (1997) Telomerase activity and its clinicopathological significance in gastric cancer. *Eur J Cancer* **33**: 1309–1313

Baba H, Korenaga D, Okamura T, Saito A and Sugimachi K (1989) Prognostic factors in gastric cancer with serosal invasion. Univariate and multivariate analyses. *Arch Surg* **124**: 1061–1064

Bando E, Yonemura Y, Takeshita Y, Taniguchi K, Yasui T, Yoshimitsu Y, Fushida S, Fujimura T, Nishimura G and Miwa K (1999) Intraoperative lavage for cytological examination in 1,297 patients with gastric carcinoma. *Am J Surg* **178**: 256–262

Bizer LS (1983) Adenocarcinoma of the stomach: current results of treatment. *Cancer* **51**: 743–745

Bodnar AG, Kim NW, Effros RB and Chiu CP (1996) Mechanism of telomerase induction during T cell activation. *Exp Cell Res* **228**: 58–64

Bonenkamp JJ, Songun I, Hermans J and van de Velde CJ (1996) Prognostic value of positive cytology findings from abdominal washings in patients with gastric cancer. *Br J Surg* **83**: 672–674

Counter CM, Gupta J, Harley CB, Leber B and Bacchetti S (1995) Telomerase activity in normal leukocytes and in hematologic malignancies. *Blood* **85**: 2315–2320

Dupont JB Jr, Lee JR, Burton GR and Cohn I Jr (1978) Adenocarcinoma of the stomach: review of 1,497 cases. *Cancer* **41**: 941–947

Gaffey MJ, Mills SE, Swanson PE, Zarbo RJ, Shah AR and Wick MR (1992) Immunoreactivity for Ber-EP4 in adenocarcinomas, adenomatoid tumors, and malignant mesotheliomas. *Am J Surg Pathol* **16**: 593–599

Harley CB (1991) Telomere loss: mitotic clock or genetic time bomb? *Mutat Res* **256**: 271–282

Hermanek P, Scheibe O, Spiessl B and Wagner G (1987) TNM classification of malignant tumors: the new 1987 edition. *Rontgen-Blatter* **40**: 200

Hiyama K, Hiyama E, Ishioka S, Yamakido M, Inai K, Gazdar AF, Piatyszek MA and Shay JW (1995a) Telomerase activity in small-cell and non-small-cell lung cancers. *J Natl Cancer Inst* **87**: 895–902

Hiyama E, Yokoyama T, Tatsumoto N, Hiyama K, Imamura Y, Murakami Y, Kodama T, Piatyszek MA, Shay JW and Matsuura Y (1995b) Telomerase activity in gastric cancer. *Cancer Res* **55**: 3258–3262

Hiyama K, Hirai Y, Kyoizumi S, Akiyama M, Hiyama E, Piatyszek MA, Shay JW, Ishioka S and Yamakido M (1995c) Activation of telomerase in human lymphocytes and hematopoietic progenitor cells. *J Immunol* **155**: 3711–3715

Hiyama E, Kodama T, Shinbara K, Iwao T, Itoh M, Hiyama K, Shay JW, Matsuura Y and Yokoyama T (1997) Telomerase activity is detected in pancreatic cancer but not in benign tumors. *Cancer Res* **57**: 326–331

© 2000 Cancer Research Campaign

Iitsuka Y, Kaneshima S, Tanida O, Takeuchi T and Koga S (1979) Intraperitoneal free cancer cells and their viability in gastric cancer. *Cancer* **44**: 1476–1480

Kim NW, Piatyszek MA, Prowse KR, Harley CB, West MD, Ho PL, Coviello GM, Wright WE, Weinrich SL and Shay JW (1994) Specific association of human telomerase activity with immortal cells and cancer. *Science* **266**: 2011–2015

Kitamura K, Beppu R, Anai H, Ikejiri K, Yakabe S, Sugimachi K and Saku M (1995) Clinicopathologic study of patients with Borrmann type IV gastric carcinoma. *J Surg Oncol* **58**: 112–117

Kodama Y, Sugimachi K, Soejima K, Matsusaka T and Inokuchi K (1981) Evaluation of extensive lymph node dissection for carcinoma of the stomach. *World J Surg* **5**: 241–248

Kodera Y, Yamamura Y, Torii A, Uesaka K, Hirai T, Yasui K, Morimoto T, Kato T and Kito T (1996) Postoperative staging of gastric carcinoma. A comparison between the UICC stage classification and the 12th edition of the Japanese General Rules for Gastric Cancer Study. *Scand J Gastroenterol* **31**: 476–480

Kodera Y, Yamamura Y, Shimizu Y, Torii A, Hirai T, Yasui K, Morimoto T and Kato T (1999) Peritoneal washing cytology: prognostic value of positive findings in patients with gastric carcinoma undergoing a potentially curative resection. *J Surg Oncol* **72**: 60–64

Koga S, Kaibara N, litsuka Y, Kudo H, Kimura A and Hiraoka H (1984) Prognostic significance of intraperitoneal free cancer cells in gastric cancer patients. *J Can Res Clin* **108**: 236–238

Langford LA, Piatyszek MA, Xu R, Schold SC Jr and Shay JW (1995) Telomerase activity in human brain tumours. *Lancet* **346**: 1267–1268

Latza U, Niedobitek G, Schwarting R, Nekarda H and Stein H (1990) Ber-EP4: new monoclonal antibody which distinguishes epithelia from mesothelia. *J Clin Pathol* **43**: 213–219

Nakanishi H, Kodera Y, Torii A, Hirai T, Yamamura Y, Kato T, Kito T and Tatematsu M (1997) Detection of carcinoembryonic antigen-expressing free tumor cells in peritoneal washes from patients with gastric carcinoma by polymerase chain reaction. *Jpn J Cancer Res* **88**: 687–692

Oka M, Hirazawa K, Yamamoto K, Iizuka N, Hazama S, Suzuki T and Kobayashi N (1996) Induction of Fas-mediated apoptosis on circulating lymphocytes by surgical stress. *Ann Surg* **223**: 434–440

Ordonez NG (1998) Value of the Ber-EP4 antibody in differentiating epithelial pleural mesothelioma from adenocarcinoma. The MD Anderson experience and a critical review of the literature. *Am J Clin Pathol* **109**: 85–89

Piatyszek MA, Kim NW, Weinrich SL, Hiyama K, Hiyama E, Wright WE and Shay JW (1995) Detection of telomerase activity in human cell and tumors by a telomeric repeat amplification protocol (TRAP). *Methods Cell Sci* **17**: 1–15

Rhyu MS (1995) Telomeres, telomerase, and immortality. *J Natl Cancer Inst* **87**: 884–894

Saji M, Westra WH, Chen H, Umbricht CB, Tuttle RM, Box MF, Udelsman R, Sukumar S and Zeiger MA (1997) Telomerase activity in the differential diagnosis of papillary carcinoma of the thyroid. *Surgery* **122**: 1137–1140

Schofield K, D'Aquila T and Rimm DL (1997) The cell adhesion molecule, E-cadherin, distinguishes mesothelial cells from carcinoma cells in fluids. *Cancer* **81**: 293–298

Schott A, Vogel I, Krueger U, Kalthoff H, Schreiber HW, Schmiegel W, Henne-Bruns D, Kremer B and Juhl H (1998) Isolated tumor cells are frequently detectable in the peritoneal cavity of gastric and colorectal cancer patients and serve as a new prognostic marker. *Ann Surg* **227**: 372–379

Suzuki T, Ochiai T, Hayashi H, Hori S, Shimada H and Isono K (1999) Peritoneal lavage cytology findings as prognostic factor for gastric cancer. *Semin Surg Oncol* **17**: 103–107

Tahara H, Nakanishi T, Kitamoto M, Nakashio R, Shay JW, Tahara E, Kajiyama G and Ide T (1995*a*) Telomerase activity in human liver tissues: comparison between chronic liver disease and hepatocellular carcinomas. *Cancer Res* **55**: 2734–2736

Tahara H, Kuniyasu H, Yokozaki H, Yasui W, Shay JW, Ide T and Tahara E (1995*b*) Telomerase activity in preneoplastic and neoplastic gastric and colorectal lesions. *Clin Cancer Res* **1**: 1245–1251

Watson JD (1972) Origin of concatemeric T7 DNA. *Nature* **239**: 197–201

Wright WE, Shay JW and Piatyszek MA (1995) Modifications of a telomeric repeat amplification protocol (TRAP) result in increased reliability, linearity and sensitivity. *Nucleic Acids Res* **23**: 3794–3795

Yashima K, Vuitch F, Gazdar AF and Fahey TJ 3rd (1997) Telomerase activity in benign and malignant thyroid diseases. *Surgery* **122**: 1141–1145

*© 2000 Cancer Research Campaign*
